# Torque Teno Virus plasma level as novel biomarker of retained immunocompetence in HIV-infected patients

**DOI:** 10.1007/s15010-020-01573-7

**Published:** 2021-02-03

**Authors:** L. Schmidt, B.-E. O. Jensen, A. Walker, V. Keitel-Anselmino, V. di Cristanziano, M. Böhm, E. Knops, E. Heger, R. Kaiser, A. de Luca, M. Oette, D. Häussinger, J. Timm, A. Fuchs, N. Lübke

**Affiliations:** 1 Institute of Virology, University Hospital Düsseldorf, Heinrich-Heine-University, Düsseldorf, Germany; 2 Department of Gastroenterology, Hepatology and Infectious Diseases, University Hospital Düsseldorf, Heinrich-Heine-University, Moorenstr. 5, 40225 Düsseldorf, Germany; 3 Institute of Virology, University of Cologne, Cologne, Germany; 4 Infectious Diseases Unit, University of Siena, Siena, Italy; 5 Department of General Medicine, Gastroenterology and Infectious Diseases, Augustinerinnen Hospital, Cologne, Germany; 6 Department of Infectious Diseases, Tropical Medicine, Nephrology and Rheumatology, Hospital St. Georg, Leipzig, Germany

**Keywords:** HIV infection, Torque Teno Virus, Immune recovery, Predictive marker, Individualized treatment

## Abstract

**Purpose:**

To predict the course of immune recovery (IR) in HIV-1-infected patients after initiation of combined antiretroviral therapy (cART) by determination of the plasma concentration of Torque Teno Virus (TTV).

TTV has been identified as marker for risk assessment in immunosuppressed patients after transplantation procedures. Here, TTV was analyzed in HIV-1-infected therapy-naïve patients to evaluate its use as predictor of the course of IR for guidance of individualized treatment.

**Methods:**

TTV DNA was quantified in plasma samples of 301 therapy-naïve HIV-1-infected patients and correlated to CD4^+^ cell count, HIV viral load, presence of the herpes viruses CMV, EBV and HHV-8, age and sex. Patients were classified according to their initial CD4^+^ cell count and to the extent of CD4^+^ T-cell increase within the first year of cART.

**Results:**

TTV DNA was detectable in 96% of the patients’ plasma samples with a median TTV plasma concentration of 5.37 log_10_ cop/ml. The baseline CD4^+^ cell count was negatively correlated with TTV plasma concentration (*p* = 0.003). In patients with a CD4^+^ cell recovery < 50 cells/µl, the median TTV plasma concentration was significantly higher compared to patients with a CD4^+^ cell recovery of > 200 CD4^+^ cells/µl (5.68 log_10_ cop/ml versus 4.99 log_10_ cop/ml; *p* = 0.011). TTV plasma concentration in combination with baseline CD4^+^ cell count were significantly correlated to CD4^+^ cell recovery (*p* = 0.004). For all other parameters considered, no significant correlation for CD4^+^ cell recovery was found.

**Conclusion:**

Within the cohort, the significantly elevated TTV plasma concentration in patients with diminished CD4^+^ cell recovery indicates a more profound immune defect. Baseline TTV plasma concentrations and CD4^+^ cell count are predictive for the course of immune recovery in HIV-1-infected patients with severe immunodeficiency.

**Supplementary Information:**

The online version contains supplementary material available at 10.1007/s15010-020-01573-7.

## Introduction

HIV infection can be effectively treated leading to life expectancy comparable to the non-infected population [1]. Despite excellent therapeutic options for suppression of viral replication, a functional cure is not expected in the near future. Thus, effective combination antiretroviral therapy (cART) remains the gold standard in HIV care. Although newly developed drug classes and substances are characterized by high potency and improved tolerability [2], side effects and limited therapeutic responses remain an issue.

Management of HIV infection and individualized treatment decisions are based on characteristics of the drugs (e.g., toxicities and pharmacokinetic profiles), the virus (e.g., viral drug resistance profile and tropism) and the patient (e.g., drug adherence, antiretroviral drug history, interactions, comorbidities, coinfections and individual differences in immune control) [3, 4]. Accordingly, multiple factors need to be considered and collectively illustrate the complexity of individualized HIV therapy. While the characteristics of drugs and the virus are well studied and mostly considered in treatment options [5, 6], host characteristics are less well understood [7].

During HIV-infection, immunodeficiency occurs and, if left untreated, finally leads to AIDS. The routine diagnostic evaluation comprises mainly of viral load measurement and the determination of the number of CD4^+^ T-cells. In absence of cART, the viral load in each patient reaches an individual set point and the time until CD4^+^ T-cells decline differs individually. While the HIV viral load is considered as a marker of the driving force of immunodeficiency, the CD4^+^ T-cell count reflects the degree of deficiency that has occurred. However, a reliable prediction of the extent and speed of recovery of the immune system after initiation of cART is not possible to date and estimates are based on the patient's current condition and the clinical experience of the practitioner. The possibility to predict the course of immune recovery (IR) could be helpful for guidance of individualized treatment concepts and risk assessment. This guidance could be applied, for example, before initiation of therapy with a two drug regimen, to monitor induction-maintenance treatment strategies, to support decisions about prophylactic treatment, to reduce drug toxicities or to decrease the risk of the development of an immune reconstitution inflammatory syndrome (IRIS) in specific risk groups by sequential or later treatment initiation [8]. We hypothesised that the Torque Teno Virus (TTV) plasma level can be used for prediction of the course or the degree of IR upon initiation of cART supporting individualized treatment strategies.

The Torque Teno virus is a small virus with a single stranded negative-sense circular DNA genome from the family of Anelloviridae and is the most abundant component of the human total blood virome. TTV is highly prevalent in humans, but to date without causal evidence for an association to specific clinical diseases [9, 10]. The reservoir of TTV replication is not fully understood, but hematopoietic stem cells and activated peripheral blood mononuclear cells seem to be involved [11–13]. TTV DNA in plasma is detectable in up to > 90% of healthy people [10, 14] with viral loads between 3 and 6 log_10_ copies/ml. The virus concentration in blood reflects the interplay between TTV replication and antiviral immune response, with an estimated daily clearance rate of more than 90% of virions [14–17].

Up to now only a few smaller studies have been performed on TTV in HIV-infected patients. In this group of patients, a higher frequency of positive TTV-DNA plasma samples than in healthy blood donors and an inverse correlation between TTV plasma levels and the CD4^+^ T-cell count were described [18, 19]. In turn, progression towards AIDS leads to increased TTV plasma concentration in tissues of HIV infected patients [20]. In addition, successful cART with improved immune function led to a decrease of the TTV DNA plasma level [21]. Although TTV replication was detected in all studied HIV patients with efficient cART, no correlation was found between the level of TTV viremia or genotypes and the level of persistent T-cell activation [22, 23].

The idea to evaluate TTV as a predictive marker for the course of IR in HIV-infected patients is based on investigations performed in the context of organ and stem cell transplantation. In the context of HSCT, TTV has proven its potential as a predictive biomarker for acute graft-versus-host disease (GVHD) and as marker for functional immune competence [24, 25]. In solid organ transplant recipients, a correlation between TTV plasma levels and the intensity of immunosuppression, signs of transplant rejection and the occurrence of infection periods was shown [26–28]. In contrast, in patients following allogeneic hematopoietic stem cell transplantation (HSCT) TTV plasma levels themselves are not predictive for immune-related outcomes and clinical events, probably due to the complex and dynamic processes in the hematopoietic and immune systems after HSCT [25]. Since HIV infection is also leading to immunosuppression with an increased risk of opportunistic infections including reactivation of persistent viruses with immunosuppressive activities such as the herpesviruses *Cytomegalovirus* (CMV), *Epstein-Barr virus* (EBV), and *Human Herpesvirus 8* (HHV-8), the speed of IR after initiation of cART is an important information. However, its course is largely unpredictable to date.

Thus, the aim of the study was to evaluate the possible utilization of TTV plasma levels in HIV-infected patients for prediction of IR during cART assessed by CD4^+^ cell increase by measurement of TTV-DNA in blood plasma of asymptomatic therapy-naïve HIV-1 infected patients and correlation of the TTV plasma level with immunological, virological and biophysical parameters.

## Methods

### Study population

The analysis of TTV plasma level was performed from long-term stored blood samples of patients included in the RESINA cohort. The RESINA study is an ongoing prospective multicenter investigation with the primary goal of continuous surveillance of transmitted HIV drug resistance in therapy-naïve HIV-1 infected patients before initiation of antiretroviral therapy [29]. In total, 38 study centers (outpatient clinics and doctor’s offices providing specialized HIV care) from North Rhine-Westphalia, the most populous federal state in Germany, and adjacent cities in Rhineland Palatinate, a neighboring federal state are contributing to the study which currently includes 4815 patients. Inclusion criteria for the RESINA study are documented HIV-infection, eligibility for cART and the agreement between treating physician and patient to start treatment. Exclusion criteria are prior exposure to antiretroviral drugs and unwillingness to participate. Information on CD4^+^ T-cell count and HIV plasma concentration used for interpretation of study data were available from the documented cohort information. All plasma samples collected within the RESINA cohort are stored long-term at − 80 °C to ensure sufficient stability of the viral nucleic acids [30–33].

### Plasma sample selection and classification

The samples for this study were retrospectively selected according to the following inclusion criteria (1) cART start without AIDS event before or within the first 3 months after start of therapy, (2) CD4^+^ T-cell count < 500/μl at start of cART and (3) HIV viral load < 200 copies/ml without virological failure defined as HIV viral load ≥ 200 cop/ml in two successive check-ups within the first 2 years after therapy initiation.

Of a total of 4815 therapy-naïve HIV-1-infected patients in the RESINA cohort, 364 fulfilled the inclusion criteria. Plasma samples for study investigations were available in 301 of those patients. These 301 patients were included in the study and classified into 3 groups according to the patients’ CD4^+^ cell recovery within the first year on cART (< 50, 50–200 and > 200 CD4^+^ cells/µl). This classification was chosen to differentiate between possible clinical courses with sufficient immune reconstitution resulting in very low risk for opportunistic infections after 1 year (CD4^+^ cell increase > 200/µl), possible persistence of a relevant immune deficiency (CD4^+^ cell increase 50–200/µl) and poor immune reconstitution with only minor change in CD4^+^ cell count (CD4 cell increase < 50/µl) [34].

For clinical applicability, the data were additionally grouped according to the initial immune status, measured by CD4^+^ cell count. The samples were divided into four subgroups (< 100, 100–200, 201–350 and > 350 CD4^+^ cells/µl) based on the initial CD4^+^ cells. The stratification is based on the differentiation of patients with a good to moderate immune status (> 350 cells CD4^+^ cells/µl) and the so-called late presenters (< 350 CD4^+^ cells/µl). The late presenters were further divided according to the CD4^+^ thresholds for opportunistic infections (Table 2) [34, 35].

To investigate a possible influence of the presence of different herpesviruses on TTV plasma concentration, the plasma concentrations of CMV, EBV and HHV-8 were determined from all participants, if an adequate sample was available (283/301; 94%).

### Virus quantification

The DNA extraction of the EDTA plasma samples was performed automatically using the Bio-Robot EZ1 with the EZ1^®^ Virus Mini Kit v2.0 (Qiagen, Hilden, Germany) according to the manufacturer’s recommendations.

The TTV-DNA was quantified by real-time PCR (qPCR) as previously described by Maggi et al. [36]. TTV standards were kindly provided by the Department of Virology of the Medical University of Vienna, Austria and stabilized for valid quantification results in the Institute of Virology of Cologne, Germany. Quantification was validated in a collaborative trial.

Quantification of CMV and EBV was performed as described by Schönberger et al. [37] and qPCR of HHV-8 was performed by amplification of a fragment of the HHV-8 ORF26 gene region using the TaqMan Universal Master Mix (Applied Biosystems).

### Statistical analyses

The determined TTV DNA plasma level presented a high range in the analyzed cohort. For this reason, the unit of copies per milliliter was changed to log_10_ values to allow differences in the analyzed groups to be captured in diagrams. Using TTV plasma level by log_10_ scale, the status of the Kolmogorov–Smirnov test presented a *p* value greater 0.05 (*p* = 0.09), so that a Gaussian distribution can be assumed, allowing parametric tests. *T*-test and Mann–Whitney-*U*-Test were used for assessment of a possible selection bias caused by the selection process of study participants depending on sample availability.

A Pearson correlation was used to compare TTV plasma levels, CD4^+^ cell counts and presence of the *Herpesviridae* CMV, EBV, HHV-8 using a 95% confidence interval (CI). In order to analyze the differences between the three groups based on the CD4^+^ T-cell recovery within the first year ANOVA tests were performed and post hoc tests were applied [Tukey’s honest significant difference (HSD)]. In addition, ordinary linear and multiple linear regressions were used to analyze the correlation of sex, age, the presence of the *Herpesviridae* CMV, EBV, HHV-8, and the plasma concentration of HIV and TTV to CD4^+^ T-cell recovery. For all statistical analyses, *p* values < 0.05 were considered significant.

The statistical analyses were performed using SPSS Statistics 25 (IBM Corp. Released 2017. IBM SPSS Statistics for Macintosh, Version 25.0. Armonk, NY, USA).

## Results

According to inclusion criteria, 364 suitable patients where identified within the RESINA cohort. Of those, a stored plasma sample for study investigations was available in 82.7% (301/364). Comparing the mean values of the parameters considered in the two groups of patients with available and unavailable plasma samples, in which individual outliers in the distribution are also taken into account, only a difference regarding the CD4^+^ cell gain of patients was seen (178 cells/µl vs. 253 cells/µl, *p* = 0.015; Supplemental Table 1), whereas age, HIV-RNA and CD4^+^ cell count at baseline were comparable. However, when using median values, which are less influenced by outliers, this analysis showed no significant differences for all these parameters including CD4^+^ cell gain.

Within the cohort of 301 patients with available plasma samples, 78% were male and the median age was 49 years (range 25–92 years, SD = 11.8 years) (Table 1). The median HIV viral load before initiation of cART was 48,394 copies/ml with an individual range between 40 and 8,858,100 copies/ml. According to the patients’ CD4^+^ cell recovery within the first year on cART and the resulting sustained risk of opportunistic infections, they were classified into three groups [< 50 (*n* = 67), 50–200 (*n* = 115) and > 200 CD4^+^ cells/µl (*n* = 119), (Table 2)].

**Table 1:** Patients’ characteristics Physiological and clinical characteristics of the patients at the time point of sample collection (*n* = 301)

Category	*N*	%
Age (median, years)	49 (range 25–92)	
Sex		
Male	234	78
Female	67	22
HIV-RNA (median, cop/ml)	48,394 (range 40–8,858,100)	
TTV-DNA (median, log_10_ cop/ml)	5.36 (range 0–9.3)	
CD4^+^ cell count at baseline (cells/µl)		
< 100	89	30
100–200	38	13
201–350	119	40
> 350	55	18

**Table 2:** Overview over baseline CD4^+^ cell counts and their gain after initiation of cART in the analysed cohort

CD4^+^ cell count at baseline (cells/µl)	CD4^+^ cell recovery (cells/µl)			
CD4^+^ cell count	< 50	50–200	> 200	∑
< 100	11	41	37	89
100–200	10	14	14	38
201–350	22	48	49	119
> 350	24	12	19	55
∑	67	115	119	301

In addition, the patients were stratified according to their initial CD4^+^ cell count to allow for a clinically oriented classification: Patients with a CD4^+^ cell count > 350 cells/µl (*n* = 55), who had a good or only moderately compromised immune status, and patients with a more pronounced immune deficiency, the so-called late presenters with a CD4^+^ cell count < 350 cells/µl. These patients were additionally classified into subgroups according to their risk for opportunistic infections: < 100 (*n* = 89), 100–200 (*n* = 38) and 201–350 cells/µl (*n* = 119) (Table 2).

**Fig. 1:**
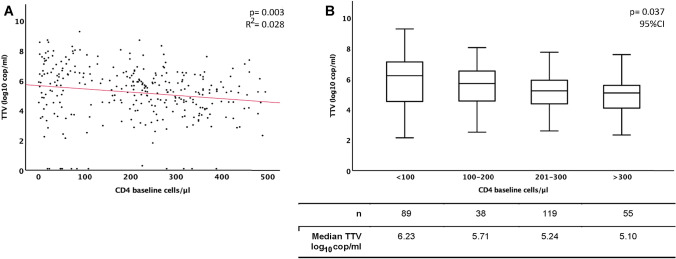
TTV plasma level correlation with baseline CD4^+^ cell count Correlation between TTV-DNA plasma concentration and the CD4^+^ cell counts before initiation of cART; **a** regression analysis of log_10_ TTV cop/ml to baseline CD4^+^ cells/µl; **b** correlation of log_10_ TTV cop/ml to specific defined strata of baseline CD4^+^ cells/µl

TTV was detected in 96.0% (289/301) of the analyzed plasma samples. The median TTV plasma concentration was 5.37 log_10_ copies/ml with a wide range between 0 and 9.30 log_10_ copies/ml. The majority of patients presented with a baseline CD4^+^ cell count between 201 and 350 (*n* = 119; 40%, Table 1). Although the regression analysis of TTV plasma levels and baseline CD4^+^ cell counts showed a wide scattering of the TTV values, it also shows a significant negative correlation, with TTV plasma concentration being increased in samples from patients with lower CD4^+^ cell counts (*p* = 0.003; *R*^2^ = 0.028, Fig. 1a). This is also confirmed by correlation of the TTV plasma level to the different CD4^+^ strata groups (Fig. 1b) which illustrates the significant negative correlation between the two parameters (*p* = 0.037). In detail, the correlation is particularly evident in the decline in median TTV viral loads with increasing CD4^+^ cell count at baseline.

**Fig. 2:**
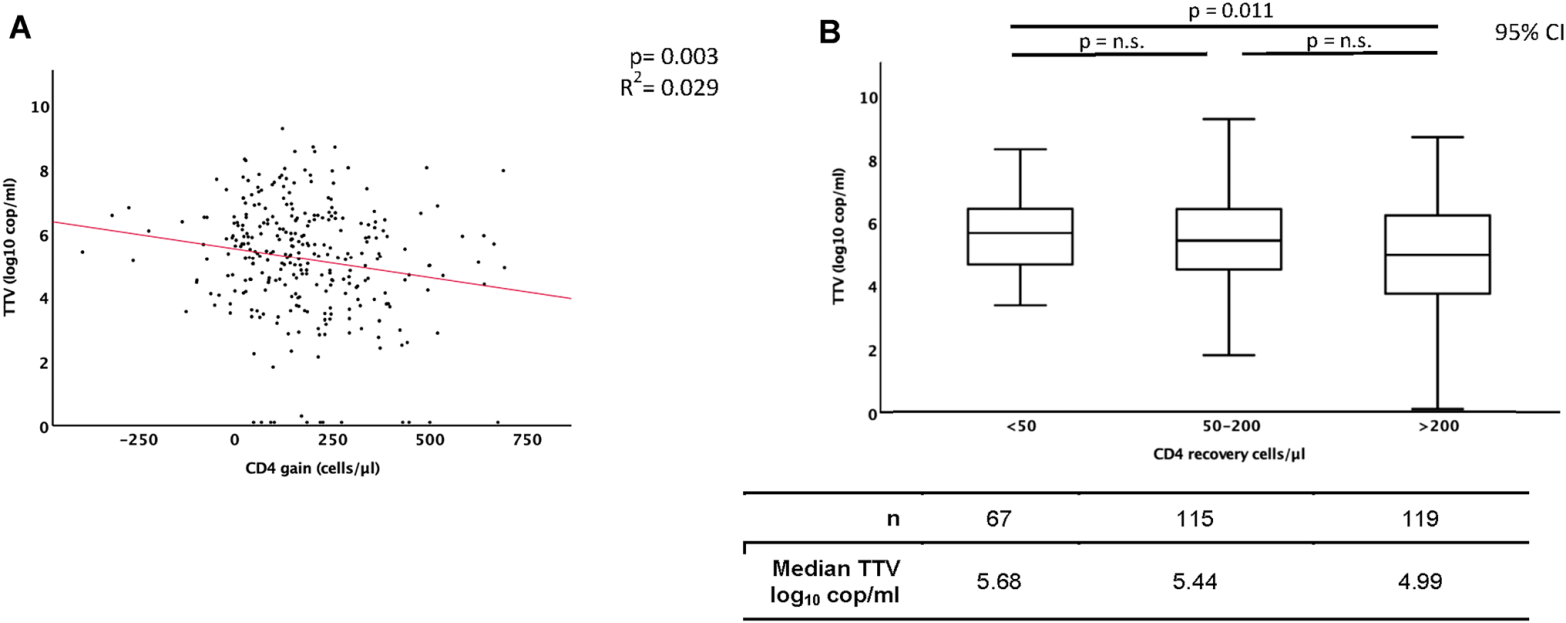
TTV plasma level correlation with CD4^+^ cell recovery Correlation between TTV-DNA plasma concentration (log_10_ cop/ml) and the CD4^+^ cell recovery within the first year of cART; **a** regression analysis of log_10_ TTV cop/ml to gain of CD4^+^ cells/µl; **b** correlation of log_10_ TTV cop/ml to CD4^+^ cells/µl stratified in a gain of < 50, 50–200 and > 200 cells/µl within the first year of cART. n.s.: not significant

To evaluate the potential of TTV plasma concentration as predictive marker for immune recovery, the TTV plasma levels were correlated to the CD4^+^ recovery within the first year on cART. The regression analysis shows a significant negative correlation, with patients with a poor immune recovery showing significantly higher TTV viral loads than those with a good one (*p* = 0.003; *R*^2^ = 0.029, Fig. 2a). Looking at the regression based on the stratified groups of CD4^+^ recovery, there are clear differences between the groups (Fig. 2b). Patients with poor recovery of CD4^+^ cells (< 50 CD4^+^ cells/µl) showed TTV viremia in 100%. In addition, the TTV plasma levels of patients with CD4^+^ recovery < 50 CD4^+^ cells/µl were significantly higher compared to patients with CD4^+^ recovery > 200 cells/µl (median 5.68 log_10_ cop/ml versus 4.99 log_10_ cop/ml; *p* = 0.011). Thus, higher TTV plasma concentration before treatment initiation was significantly correlated to lower CD4^+^ gain within the first year on cART.

Regarding the detection of herpesvirus DNA, adequate samples of 283/301 (94.0%) study participants were available. CMV, EBV and HHV-8 were detected in 35/283 (12.4%), 74/283 (26.1%), and 29/283 (10.2%) samples, respectively. EBV and HHV-8 were not correlated with the initial immune status, whereas CMV DNA was significantly more frequently detected in patients with CD4^+^ cell counts < 100 cells/µl (Supplementary Table 2). Furthermore, also the extent of CMV plasma viral load was significantly inversely correlated with the initial CD4^+^ cell count (*p* < 0.001; Supplementary Fig. 1). A significant correlation of CMV, EBV or HHV-8 detection to TTV prevalence was not observed (*p* = 0.085, *p* = 0.652, *p* = 0.277, respectively; Supplementary Table 3).

For further evaluation of TTV plasma concentration as predictive marker for CD4^+^ recovery, regression analyses including the parameters sex, the presence of CMV, EBV and HHV-8 DNA and CD4^+^ baseline cell count were performed (Table 3). Univariate regression analysis indicated no correlation of sex or detection of CMV, EBV and HHV-8 DNA with CD4^+^ T-cell recovery, but a significant correlation between TTV plasma baseline CD4^+^ values < 100 cells/µl and > 350 cells/µl and CD4^+^ T-cell recovery within the first year on therapy (OR 0.383, 95%CI 0.190–0.773, *p* = 0.006 and OR 3.554, 95%CI 1.903–6.639, *p* < 0.001, respectively).

**Table 3:** Univariate regression analysis for parameters associated with CD4^+^ cell recovery within the first year of cART

Variable	*N*	Univariate analysis	
		OR (95% CI)	*p* value
Total participants	301		
CD4^+^ cell count at baseline (cells/µl)			
< 100	89	0.383 (0.190–0.773)	0.006*
100–200	38	1.262 (0.579–2.750)	0.559
201–350	119	0.729 (0.414–1.285)	0.275
> 350	55	3.554 (1.903–6.639)	< 0.001*
Sex			
Male	234	0.915 (0.816–1.107)	0.499
Female	67	1.184 (0.732–1.915)	0.503
CMV DNA positive	35	1.282 (0.506–3.252)	0.600
EBV DNA positive	74	1.019 (0.527–1.968)	0.956
HHV-8 DNA positive	29	0.790 (0.320–1.950)	0.608

Odds ratios (ORs) and 95% confidence intervals (CIs) for the selected parameters CD4^+^ cell count at baseline, sex and CMV, EBV and HHV8 DNA detection in correlation to CD4^+^ cell recovery within the first year of cART (*: statistically significant correlation)

Considering all variables in the multiple linear regression analysis, no association of HIV RNA, sex and age, but a significant correlation between the combination of TTV plasma levels and baseline CD4^+^ values and CD4^+^ recovery within the first year on cART was observed (*p* = 0.001 and *p* = 0.006, respectively; Table 4). In summary, a prediction of the CD4^+^ gain within the first year of cART was possible by determining TTV plasma level and CD4^+^ cell count at baseline (adjusted *R*^2^ = 0.042) [38].

**Table 4:** Multiple linear regression analysis for parameters associated with CD4^+^ cell recovery within the first year of cART

	*B*	SD	*ß*	*p* value	Cumulative adjusted *R*^2^
TTV DNA (log_10_ cop/ml)	− 19.271	5.550	− 0.203	0.001*	
HIV RNA (cop/ml)	− 8.449E-7	< 0.001	− 0.003	0.953	
CD4^+^ cell count at baseline (cells/µl)	− 0.203	0.073	− 0.165	0.006*	
Sex	4.337	23.493	0.011	0.854	
Age	0.091	0.843	0.006	0.914	
Cumulative adjusted *R*^2^					0.042

Non-standardized regression coefficient (*B*), standard deviation (SD), standardized regression coefficient beta (*ß*) and *p* value. The dependent variable was CD4^+^ gain within the first year on cART. Independent variables were TTV DNA log_10_ plasma level, HIV viral load, CD4^+^ cell count at baseline, sex and age (*: significant correlation). Overall final model (5, 293) = 3.584, *p* = 0.004, *R*^2^ = 0.058, adjusted *R*^2^ = 0.042

Collinearity between CD4^+^ cell count and TTV plasma level could be excluded with a variance inflation factor (VIF) of 1.038 for TTV and a VIF of 1.121 for CD4^+^ cells at baseline. For all other parameters considered, no significant correlation for CD4^+^ cell recovery was found.

## Discussion

Currently, routine state-of-the-art monitoring of the extent of immune deficiency caused by an HIV infection usually relies on surveillance of surrogate parameters, most commonly CD4^+^ cell count, but more comprehensive determination of immune status and prediction of IR are difficult. The possibility to further assess immunocompetence and predict the course of IR after initiation of cART would help to guide individualized treatment. Thus, the plasma level of TTV DNA in plasma samples of HIV-1 infected therapy-naïve patients was evaluated for its applicability as novel parameter for the prediction of IR for improved management of personalized HIV treatment concepts.

In our study, 96% of the patients’ plasma samples showed evidence for active TTV replication, confirming a high prevalence of TTV in HIV-infected patients [39]. This provides the necessary basis of TTV being a possible predictive marker for IR in HIV-infected therapy-naïve patients commencing cART. The median viral load of TTV in this cohort was 5.37 log_10_ copies/ml and is comparable to that of other high-risk cohorts [26, 39, 40], but the plasma concentration is characterized by a wide range, namely between 0 and 9.30 log_10_ copies/ml. This high variation of the TTV plasma level in HIV-infected patients as well as the negative correlation of the TTV plasma level and the baseline CD4^+^ cell count confirms the observations of previous studies [18, 20, 41].

The correlation analysis between TTV plasma level at baseline and CD4^+^ cell recovery within the first year of cART revealed significantly higher TTV loads in patients with a poor recovery (< 50 CD4^+^ cells/µl) compared to patients with a good recovery (> 200 CD4^+^ cells/µl). There was no influence of CMV, EBV and HHV-8 on TTV plasma level, as also seen in immunosuppressed patients after hematopoietic stem cell transplantation [42]. This indicates the potential of TTV as predictive marker for IR in HIV-1-infected patients.

Ordinary and multiple linear regression analyses including the parameters sex, age, HIV viral load, TTV plasma level, detection of the *Herpesviridae* CMV, EBV or HHV-8 and CD4^+^ baseline values have excluded the parameters sex, age, HIV viral load and the prevalence of herpesvirus DNA as valuable parameters for prediction of CD4^+^ cell recovery. The fact that age has no significant influence on immune reconstitution was initially surprising, as several studies have shown that immune recovery decreases with age [43–45]. Other studies confirm our observation that there is no significant correlation between age and immune recovery [46]. However, it is important to consider the age distribution in our cohort. Although a wide range is noticeable, the two quartiles from 25 to 75% only cover 41.5–56 years (median: 49 years), with a standard deviation of 11.8 years. This indicates only a small age distribution, which may be one of the reasons why no correlation was found in this study. The observation that detection of the various herpes viruses had no significant effect on immune recovery is also an important finding that favors the predictive value of TTV plasma concentration for immune recovery in HIV-infected patients, as our analysis revealed that the combination of TTV plasma level and the initial CD4^+^ cell count was predictive for IR in our cohort.

Although TTV plasma concentration alone showed also a significant correlation to immune recovery, its predictive power was not sufficient to classify IR in defined ordinal scales (< 50, 50–200 and > 200 CD4^+^ cells/µl), as also seen in other studies [22, 23]. The combination of TTV plasma concentration and initial CD4^+^ cell count still did not accurately predict the extent of IR, as indicated by the low determination coefficient [47], but the combined predictive power of both variables seems sufficient to provide some guidance to clinicians to predict a good vs. a poor IR, as demonstrated in our analyses.

Although a large data set was available to select suitable patients for this study, not all of the suitable patients had appropriate stored plasma samples available for study investigations (301/364). In particular, 9/76 (11.8%) patient samples of group A (IR < 50 CD4^+^ cells/µl), 25/140 (17.9%) of group B (IR 100–200 CD4^+^ cells/µl) and 29/148 (19.6%) of group C (IR > 200 CD4^+^ cells/µl) were unavailable. The fact that a plasma sample was not available from all patients who met the inclusion and exclusion criteria for this analysis leads to a risk of a selection bias. When comparing the two groups of patients meeting the inclusion criteria with and without available plasma samples, no significant differences were found for the median values of the investigated parameters. Only the comparison of the mean values revealed a significant difference for the parameter immune response (CD4^+^ cell gain). After examination of the raw data, this difference could mainly be attributed to individual outliers. Therefore, we expect only a small influence of the selection bias on our results, even if it cannot be completely excluded.

One further possible limitation of this retrospective analysis is the sample distribution within the groups. Although the sample size was sufficient to ensure statistical power, there was no even sample distribution within the groups according to CD4^+^ cell gain, as a smaller number of patients with a low CD4^+^ cell gain was available to be analyzed. This distribution can be attributed to the confined number of patients with limited extent of immune reconstitution in the RESINA cohort. Other parameters possibly influencing the CD4^+^ cell recovery are the CD8^+^ cell count or the CD4^+^/CD8^+^ ratio, co-infections as Hepatitis B or Hepatitis C or the treatment regimen used to initiate cART. Unfortunately, the documentation concerning these parameters by the participating centers was quite frequently incomplete. Thus, the parameters were not analyzed systematically during this investigation. Further interesting aspects that should be considered for having a potential impact on IR are the time until viral suppression after initiation of cART or the number and frequency of blips occurring during treatment. Those parameters are to be considered during future prospective investigations to further analyze the impact of TTV plasma level as predictive marker for IR.

In conclusion, the high prevalence of TTV-DNA in blood samples of HIV-1-infected patients provides the necessary precondition for it being used as predictive marker for the recovery of immune function. In this cohort, there was a significant correlation between the baseline TTV plasma level in combination with the CD4^+^ cell count and the course of IR. The combination of a high TTV plasma concentration and low number of CD4^+^ cells before treatment initiation was associated with poor IR within the first year of cART. The determination of the baseline CD4^+^ cell count alone did not predict immune reconstitution.

This study demonstrates the potential of TTV plasma load as a predictive marker of the course of immune reconstitution and as an additional surrogate parameter potentially allowing a more detailed interpretation of retained or lost immunocompetence in HIV-1-infected patients compared to the CD4^+^ cell count alone. In combination with the baseline CD4^+^ cell count, the TTV DNA plasma level enables clinicians to predict the course of immune reconstitution, which could help to provide guidance for individualized treatment decisions as for example planning of indication and duration of prophylactic anti-infective treatments to prevent opportunistic infections, to avoid unnecessary drug toxicity or before initiating glucocorticoids in tuberculosis patients with high risk of IRIS [48].

## Supplementary Information

Below is the link to the electronic supplementary material.

Supplementary file1 (DOCX 18 KB)

Supplementary file2 (DOCX 15 KB)

Supplementary file3 (DOCX 13 KB)
