# Supplementary material for: Torque Teno Virus plasma level as novel biomarker of retained immunocompetence in HIV-infected patients
Source: Infection. 2021 Feb 3;49(3):501–9. doi: 10.1007/s15010-020-01573-7 (PMC8159784; doi:10.1007/s15010-020-01573-7)
Supplement: Supplementary file 1 — Supplementary file1 (DOCX 18 KB) [file 15010_2020_1573_MOESM1_ESM.docx]

**Supplemental Table 1: Baseline characteristics of patients with fulfilled inclusion criteria and available plasma sample vs. patients with fulfilled inclusion criteria and no available plasma sample** (*: significant difference)

1. Physiological and clinical characteristics (median) of the patients and p-values of Median test for independent samples

| **Baselines characteristics** | **Total** | **available** | **unavailable** | **p-value** |
| --- | --- | --- | --- | --- |
| Number of samples (n) | 364 | 301 | 63 |  |
| Age (years) | 50 | 49 | 51 | 0.348 |
| HIV RNA (cop/ml) | 47,810 | 48,394 | 43,692 | 0.981 |
| CD4^+^ cell count at baseline (cells/µl) | 222 | 225 | 219 | 0.782 |
| CD4^+^ cell gain (cells/µl) | 166 | 158 | 188 | 0.064 |

1. Physiological and clinical characteristics (mean) of the patients and p-values of T-test for independent samples

| **Baseline characteristics** | **Total** | **available** | **unavailable** | **p-value** |
| --- | --- | --- | --- | --- |
| Number of samples (n) | 364 | 301 | 63 |  |
| Age (years) | 50.2 | 49.5 | 50.7 | 0.376 |
| HIV RNA (cop/ml) | 206,115 | 214,468 | 166,338 | 0.581 |
| CD4^+^ cell count at baseline (cells/µl) | 212 | 215 | 211 | 0.794 |
| CD4^+^ cell gain (cells/µl) | 188 | 178 | 235 | **0.015*** |
