# Supplementary material for: Torque Teno Virus plasma level as novel biomarker of retained immunocompetence in HIV-infected patients
Source: Infection. 2021 Feb 3;49(3):501–9. doi: 10.1007/s15010-020-01573-7 (PMC8159784; doi:10.1007/s15010-020-01573-7)
Supplement: Supplementary file 2 — Supplementary file2 (DOCX 15 KB) [file 15010_2020_1573_MOESM2_ESM.docx]

**Supplemental Table 2: Detection of CMV, EBV and HHV-8 in therapy naïve HIV-infected patients depending on baseline CD4^+^ cell count**

| Virus | CD^+^ cells baseline (cells/µl) | | | | |  |
| --- | --- | --- | --- | --- | --- | --- |
|  | **Total**  **(n=283)** | **< 100**  **(n=85)** | **100-200**  **(n=37)** | **201-350**  **(n=115)** | **>350**  **(n=46)** | **p-value** |
| CMV | 35 (12.4%) | 29 (34.1%) | 3 (8.1%) | 1 (0.9%) | 2 (4.3%) | **<0.001*** |
| EBV | 74 (26.1%) | 16 (18.8%) | 14 (37.8%) | 33 (28.7%) | 11 (23.9%) | 0.140 |
| HHV-8 | 29 (10.2%) | 11 (12.9%) | 4 (10.8%) | 10 (8.7%) | 4 (8.7%) | 0.776 |

*: statistically significant (Chi square according to Pearson)
