# Supplementary material for: Torque Teno Virus plasma level as novel biomarker of retained immunocompetence in HIV-infected patients
Source: Infection. 2021 Feb 3;49(3):501–9. doi: 10.1007/s15010-020-01573-7 (PMC8159784; doi:10.1007/s15010-020-01573-7)
Supplement: Supplementary file 3 — Supplementary file3 (DOCX 13 KB) [file 15010_2020_1573_MOESM3_ESM.docx]

**Supplemental Table 3: Correlation of the presence of CMV, EBV and HHV8 to prevalence of TTV in plasma samples of therapy naïve HIV-infected patients**

| **Virus** | **TTV pos (n=273)** | **TTV neg (n=10)** | **p-value** |
| --- | --- | --- | --- |
| **CMV** | 32 (11%) | 3 (30%) | 0.085 |
| **EBV** | 72 (26.4%) | 2 (20%) | 0.652 |
| **HHV-8** | 29 (10.6%) | 0 (0%) | 0.277 |

P-values determined by Chi square according to Pearson
